# Cutaneous leishmaniasis a neglected tropical disease: community knowledge, attitude and practices in an endemic area, Northwest Ethiopia

**DOI:** 10.1186/s12879-019-4506-1

**Published:** 2019-10-16

**Authors:** Helina Fikre Tamiru, Yohana James Mashalla, Rezika Mohammed, Gloria Thupayagale Tshweneagae

**Affiliations:** 1 Department of Health Studies, University of South Africa, Pretoria, South Africa; 2 Leishmaniasis Research and Treatment Centre, University of Gondar, Gondar, Ethiopia; 3 Faculty of Medicine, University of Botswana, Gaborone, Botswana

**Keywords:** Cutaneous leishmaniasis, Knowledge, Attitude, Practice, Ethiopia

## Abstract

**Background:**

Cutaneous leishmaniasis is one of the neglected tropical diseases in the Ethiopian highlands and studies on assessment of knowledge, attitude and practice of the community in endemic areas are scanty. The study aimed to assess the knowledge, attitude towards cutaneous leishmaniasis and treatment seeking practices in people living in the endemic highlands areas in the Northwest, Ethiopia and to provide evidence-based information to guide development of appropriate interventions to reduce the impact of cutaneous leishmaniasis on communities.

**Methods:**

Quantitative cross-sectional study was conducted in cutaneous leishmaniasis endemic districts (woredas) using a semi structured questionnaire. Households were randomly selected according to probability proportional to size of households in each enumeration area. Systematic random sampling of eligible households was based on the number of households recorded during listing of households. Descriptive statistics was used to describe numerical data, organise and summarise the data in a manner that gave meaning to the numerical form. Frequency tables were used to show descriptive analysis and regression analysis was used to determine correlation between variables.

**Results:**

Majority of respondents 321(78.7%) lived in rural areas, age ranged between 18 and 85 years and most were farmers. Illiteracy was high (47.6%) among respondents and majority 358(87.8%) had seen patients with CL. Less than quarter (21.6%) had heard about sand flies and knowledge on the peak transmission period was low (46.3%). About 192 (47.1%) of the respondents indicated disfiguring lesions were the major clinical presentations, less than half 55(27.5%) of urban residents believed CL was treatable compared to 145(72.5%) of rural residents (*P* < 0.001). Traditional medicines were indicated as best treatment option by 209(51.2%) compared to 114(27.9%) for modern treatment. Major factors influencing treatment options included accessibility to treatment facilities, distance and short duration of treatment. Participants expressed negative experiential attitude and perceived control towards modern treatment because of inaccessibility and distance from where modern treatment is provided.

**Conclusion:**

Priority should be given to primary prevention and appropriate awareness campaigns on lesion recognition. Information on modern treatment should be intensified.

## Background

Leishmaniasis is a tropical disease caused by a vector-borne protozoan parasite of the genus *Leishmania* and transmitted by bites of infected female sandflies *(Phlebotomus and Lutzomyia)*. About 98 countries of the world are affected with estimated 350 million people being at risk. With a global prevalence of approximately 12 million people and annual incidence 2–2.5 million cases, the disease is endemic in many countries [1]. The clinical manifestation of leishmaniasis is influenced by the infected vector resulting in three distinct presentations including cutaneous leishmaniasis (CL), mucosal involvement (MCL) and systemic visceral involvement (VL) [2]. Worldwide, Cutaneous leishmaniasis is the most common form of leishmaniasis and approximately 90% occurring in the Middle East and southern America countries [3]. More than 20 types of leishmania species are responsible for CL. Immunological studies have classified Leishmania parasites into Old World species including Leishmania *major*, Leishmania *infantum* and Leishmania *tropica* that are commonly found in the Middle East, Mediterranean basin and the Horn of Africa and the New World species commonly found in the southern America countries [4].

Ethiopia, on the Horn of Africa is among the countries with a high burden of cutaneous leishmaniasis estimated to range from 20,000 to 30,000 cases per year and the disease is endemic in the highland areas with an elevation of 1400–3175 m above sea level [5]. According to a systematic review and meta-analysis of leishmaniasis in Ethiopia [6] the most dominant type of leishmaniasis is visceral leishmaniasis (VL) much more devastating in the northern part of the country [7]. The major concern regarding VL is its high fatality rate which may rise to up to 100% among non-treated compared to only 10% among treated patients [8]. Leishmania *major* has also been reported in the country [9]. Leishmania *aethiopica* is the main cause of CL in Ethiopia causing the most severe forms of CL such as diffuse CL with multiple skin lesions characterised by non-ulcerating papular, nodular and plaque involving most parts of the body [10]. Three clinical presentations of CL have been reported in the country including localised cutaneous leishmaniasis which is characterised by localised papular or nodular lesion at the site of the sand-fly bite. The ulcer is usually painless, pink, and round with well-defined raised edges and in some cases could be self-limiting. Also reported in the country is muco-cutaneous leishmaniasis characterised by mucosal (nasal, oral, pharynx, larynx) involvement either by direct bite on the mucosal surface or by extension of the localized cutaneous leishmaniasis [11]. Previous studies have indicated that disfigurement due to CL has both socially and psychologically impacts causing anxiety, stress, depression and low quality of life which ultimately impacts on individual’s economic productivity. Therefore, treating cases and limiting potential scar formation and disfigurement are recommended measures in reducing the impact of CL [12, 13].

Direct relationship between awareness of the population at risk of a disease and adoption of preventive measures has been reported [14]. Most studies on CL in Ethiopia have focused on assessing safety and effective treatment of CL and have reported that Sodium Stibogluconate (SSG) remains the first line drug of choice. The treatment outcome however, is usually poor as most patients report for treatment several months (average 19) from commencement of symptoms and treatment which require SSG injections for two or more months [15]. Few health facilities in the country that have the capacity to diagnose and treat CL with the majority located in cities far from endemic areas complicates CL health promotion and control measures in these communities. Therefore, this study aimed to assess the knowledge, attitude and practices of the communities towards CL in the endemic areas in Gondar zone with a view to provide evidence-based data that will contribute to the success of leishmaniasis prevention and control programmes.

## Methods

A quantitative, descriptive cross-sectional survey was conducted to assess the level of knowledge, attitude and practices about CL among people living in four endemic areas in northwest, Ethiopia.

### Study setting

Located on the north western and central part of Ethiopia, Amhara region consists of 7 zones and 105 districts [16]. Four districts from Northwest Ethiopia including Gondar, Lay Gayint, Maksegnit and Armachiho were selected in this study. Selection of the districts was purposively done based on the case burden registered at the Leishmaniasis Research and Treatment Centre (LRTC), Gondar University Hospital where most patients from northwest Ethiopia visit for diagnosis and treatment of both cutaneous and visceral leishmaniasis. The Centre was established in collaboration with the Drug for Neglected Diseases Initiative and University of Gondar for research and treatment of leishmaniasis. Each district had an average of 30 *kebeles* or villages and each *kebele* had an average of 700 households. Each household had an estimated population of 5 people living together.

### Study design and sampling procedures

Quantitative cross-sectional study was conducted in cutaneous leishmaniasis endemic districts using a semi structured questionnaire. The study was carried out in December 2017. Purposive sampling technique was used to select 15 *kebeles* giving a total of 10,500 accessible households. The estimated population in each *kebele* ranged from 3500 to 4000; therefore, an estimated 52,500–60,000 population was accessible in the study. Two-stage cluster sampling approach was used to determine the sample size. In the first stage, 15 sample points (Enumeration Areas, EAs) were selected independently from all the strata with Probability Proportional to Size (PPS) of households using the 2007 Population and Housing Census data [17]. Since the prevalence of CL was not known, 50% prevalence of CL was used to determine the sample size. Simple random sampling was used to select participants into the study. Both male and female adults aged 18 years and above were eligible for inclusion in the study and the calculated sample size was 384. A 6% design defect and non-response rate was added to the calculated sample size. Therefore, total of 408 participants took part in the study.

### Data collection

The data collection instrument (questionnaire) was designed by the investigator. Prior to data collection, the questionnaire was pre-tested on eight persons who had similar characteristics but were not part of the study population. Thirty (30) health extension workers were employed to administer the questionnaires after being trained on data collection procedures and ethical consideration.

### Data analysis

Descriptive statistics was used to organise, describe and synthesise the data in order to facilitate insight about knowledge, attitude and practices on CL. Inferential statistics to test relationships between the variables and demographic factors was also used and the level of significance (*p* = 0.25) as two-tailed test was employed. Epi info 7 was used for descriptive statistics and SPSS version 16 was used to further analyse the data. Regression analysis was used to determine the association between variables and frequency tables were used to show descriptive analysis results.

### Ethical consideration

Ethical clearance (Ref: HSHDC/784/2017) was obtained from the Research and Ethics Committee of the Department of Health Studies of the University of South Africa and Institutional Review Board of University of Gondar, Ethiopia (O/V/P/RCS/05/57/2017). Permission to conduct the study in the districts was obtained from the local Administrative Departments. All participants in the study were subjected to a consent form after the purpose and the procedures were clearly explained to them to a level that they comprehended and willingly consented. The names of participants were not entered on the research instrument and there was no link between the participant and the collected data.

## Results

### Sociodemographic characteristics of the study population

Four hundred eight (408) adults participated in the study; 234(57.4%) and 174 (42.7%) were female and male respectively. Majority 321(78.7%) were living in rural areas and 87 (21.3%) lived in urban areas. Age ranged between 18 and 85 years; the mean and median ages were 36 and 35–45 years respectively. Most participants 255 (62.5%) engaged in farming, 41(10.1%) were government employees and 55(13.5%) were self-employed. Illiteracy was high 194(47.6%), 21(5.2%) could read and write, 114(27.9%) and 59(14.5%) had completed primary and secondary education respectively. Only 20(4.9%) had attained higher education qualifications (Table 1).

**Table 1:** Sociodemographic characteristics of the study participants in Northwest, Ethiopia

Variable	Frequency	Percent (%)
Age		
18–30	133	32.6
31–40	140	34.3
41–50	90	22.1
51–60	31	7.6
≥ 61	14	3.4
Gender		
Female	234	57.4
Male	174	42.7
Education level		
Read and write	21	5.2
Primary	114	27.9
Secondary	59	14.5
Tertiary	20	4.9
Illiterate	194	47.6
Occupation		
Farmer	255	62.5
Government employee	41	10.1
House wife	30	7.4
Private	55	13.5
Student	12	2.9
Unemployed	15	3.7

### Knowledge about CL, the vector, clinical presentation and sources of information

Table 2 summarises participant’s knowledge about CL, the vector, clinical presentations and sources of information about CL. Most participants, 358(87.8%) had seen CL previously and 315(77.9%) recognised CL as one of the health problems in the area. CL was locally described as “*Setie*” meaning female softer swollen lesions and “*Wondie*” meaning male rough and hard lesions that do not heal easily. Almost one third 108(26.7%) described CL as a disease characterised by lesions mainly affecting the face and 57(13.9%) described it as a disease with very ugly disfiguring lesions that may even deform the nose causing disability. About 10% believed the disease is caused by bats and small percentages related the disease to lack of hygiene and punishment from God. Education did not associate significantly with the knowledge about the disease while location (rural vs urban) associated significantly (*P* < 0.025) with the knowledge about CL with rural residents being more knowledgeable about the disease than urban residents (Tables 3 and 4). Less than quarter 88(21.6%) had heard about sand fly of which 57(13.97%) and 51(12.5%) indicated that waste disposal and unhygienic places respectively were breeding sites for sand flies. Majority 237(58.1%) did not know the time when sand flies bite while 8(21.6%) and 70(17.2%) indicated at dawn/dusk and midnight respectively.

**Table 2:** Knowledge about CL among participants resident in CL endemic areas in northwest, Ethiopia

Variables	Frequency	Percent (%)
Seen a cutaneous leishmaniasis		
No	50	12.3
Yes	358	87.8
Signs of CL		
Painful lesion/disfiguring	34	8.3
Painless/disfiguring	9	2.2
Painful skin lesion	103	25.3
Disfiguring skin lesion	149	36.5
Fever	4	0.9
Other	5	1.2
I don’t know	55	13.5
Have you seen a person with CL at your vicinity?		
Yes	315	77.2
No	91	22.3
Which gender is most affected by CL?		
Both	79	19.4
Female	71	17.4
Male	135	33.1
I don’t know	121	29.7
Indicate which parts of the body are mostly affected by CL		
Face	324	79.4
I don’t know	69	16.9
No specific site	15	3.7
Which age groups are most affected by CL?		
Adult	68	16. 7
Adult/elderly	1	0.3
Children	29	7.1
Elderly	22	5.4
All age	225	55.2
I don’t know	62	15.2
How is CL transmitted?		
By mosquito bite	24	5.9
Sand fly	93	22.8
Air droplet	5	1.2
Direct contact	39	9.6
Other	110	26.9
I don’t know	137	33.6
Have you heard of sand fly?		
Yes	87	21.3
No	321	78.7
Does Sand fly transmit diseases?		
No	109	26.7
Yes	219	53.7
I don’t know	80	19.6
What types of diseases are transmitted by sandfly?		
Diarrhoea	75	18.4
Fever	26	6.4
Cutaneous leishmaniasis	153	37.5
Gastritis	4	0.9
Skin lesion	64	15.7
I don’t know the name	19	4.7
I don’t know	11	2.7
Where do sandfly breed?		
Building ruins	2	0.5
Farmland	4	0.9
In house	3	0.7
Unhygienic place	35	8.6
Waste disposal sites	36	8.8
Other	58	14.2
I don’t know	270	66.2
What time do sand flies bite?		
Dawn/dusk &midnight	31	7.6
Dawn/dusk day	7	1.7
Day/mid night	1	0.3
Dawn/dusk	48	11.8
Day	20	4.9
Mid night	41	10.1
All the time	23	5.6
I don’t know	237	58.1
Which season of the year is peak for CL?		
Summer and winter	7	1.7
Winter and spring	3	0.7
Winter & spring & summer	1	0.3
Autumn only	31	7.6
Summer and spring	2	0.5
Summer/spring/autumn	6	1.5
Spring only	36	8.8
Summer only	88	21.6
Winter only	26	6.4
All year round	19	4.7
I don’t know	189	46.3
What are your sources of information on CL?		
TV and radio	2	0.5
Radio only	9	2.2
TV only	4	1.0
Community health education	6	1.5
Community centre	225	55.2
Health education (Schools)	61	14.9
Newsletters	3	0.7
No sources of information	97	23.8
Other sources	1	0.3

**Table 3:** Association between knowledge and attitude towards CL and the education levels among residents in CL endemic areas in northwest Ethiopia

Questions/responses	Education levels frequency and percentages				χ2	*P*-value
	Primary n (%)	Secondary n (%)	Tertiary n (%)	Illiterate n (%)		
Have you ever seen patients with CL?						
No	81(6)	8 (16)	4(8)	30(60)	4.694	0.196
Yes	106(29.6)	51(14.2)	16(4.5)	185(51.7)		
Have you heard of sand fly?						
No	88(27.4)	44(13.7)	16(5)	173(53.9)	1.172	0.76
Yes	26(29.9)	15(17.2)	4(4.6)	42(48.3)		
Do sand flies transmit CL?						
No	24(22)	12(11)	5(4.6)	68(62.4)	7.42	0.284
Yes	69(31.5)	36(16.4)	10(4.6)	104(47.5)		
I don’t know	21(26.2)	11(13.8)	5(6.2)	43(53.8)		
How is CL transmitted?						
I don’t know	32(23.4)	16(11.7)	9(6.6)	80(58.4)	11.96	0.449
Direct contact and air droplets	11(25)	6(13.6)	1(2.3)	26(59.1)		
Sand fly	26(28)	18(19.4)	6(6.5)	43(46.2)		
Mosquitoes	6(25)	4(16.7)	0(0.0)	14(58.3)		
Others	39(35.5)	15(13.6)	4(3.6)	52(47.3)		
Is CL a serious disease?						
No	6(30)	1(5.0)	0(0.0)	13(65)	7.939	0.243
Yes	99(29.4)	49(14.5)	19(5.6)	170(50.4)		
I don’t know	9(17.6)	9(17.6)	1(2)	32(62.7)		
Is CL a treatable disease?						
No	31(30.4)	12(11.8)	4(3.9)	55(53.9)	11.67	0.07
Yes	58(29)	33(16.5)	15(7.5)	94(47)		
I don’t know	25(23.6)	14(13.2)	1(0.9)	66(62.3)		
Do you know any sand fly prevention methods?						
I don’t know	32(21.5)	16(10.7)	7(4.7)	94(63.1)	10.59	0.014
Yes	82(31.7)	43(16.6)	13(5)	121(46.7)		
What types of treatment for CL do you know?						
Traditional	76(26.9)	39(13.8)	13(4.6)	155(54.8)	22.93	0.028
Modern	21(52.5)	5(12.5)	3(7.5)	11(27.5)		
I don’t know	15(21.4)	11(15.7)	2(2.9)	42(60)		
Others	1(11.1)	2(22.2)	1(11.1)	5(55.6)		
What is your best treatment option?						
Modern drugs	41(36)	22(19.3)	8(7)	43(37.7)	14.59	0.024
Traditional drugs	51(24.3)	26(12.4)	9(4.3)	124(59)		
I don’t know	22(26.2)	11(13.1)	3(3.6)	48(57.1)

**Table 4:** Association between the knowledge of CL and attitude towards treatment and residence among residents in CL endemic areas in North West Ethiopia

Question/responses	Residence		χ2	*P*-value
	Urban n(%)	Rural n(%)		
Have you ever seen a patient with CL?				
No	24(48)	26(52)	23.53	0.000
Yes	64(17.9)	294(82.1)		
Have you ever heard of sand fly?				
No	73(22.7)	248(77.3)	1.224	0.269
Yes	15(17.2)	72(82.8)		
Do sand flies transmit CL?				
No	28(25.7)	81(74.3)	13.58	0.01
Yes	33(15.1)	186(84.9)		
I don’t know	27(33.8)	53(66.2)		
How is CL transmitted?				
I don’t know	43(31.4)	94(68.6)	22.8	0.008
Direct contact and air droplets	14(31.8)	30(68.2)		
Sand fly	18(19.4)	75(80.6)		
Mosquitoes	4(16.7)	20(83.3)		
Other means	9(8.2)	101(91.8)		
Is CL a serious disease?				
No	3(15)	17(85)	10.94	0.004
Yes	65(19.3)	272(80.7)		
I don’t know	20(39.2)	31(60.8)		
Is CL a treatable disease?				
No	8(7.8)	94(92.2)	15.77	0.000
Yes	55(27.5)	145(72.5)		
I don’t know	25(23.6)	81(76.4)		
Are there sand fly prevention methods?				
Yes	46(17.8)	213(82.2)		
I don’t know	42(28.2)	107(71.8)	6.079	0.014
What types of treatment for CL do you know?				
Traditional medicines only	49(17.3)	234(82.7)	19.12	0.001
Modern medicines only	7(17.5)	33(82.5)		
I do not know	24(34.3)	46(65.7)		
Both traditional and modern medicines	3(50)	3(50)		
Others	5 (55.6)	4(44.4)		
What is your best treatment option?				
Modern medicines	29(25.4)	85(74.6)	9.636	0.008
Traditional medicines	33(15.7)	177(84.3)		
I don’t know	26(31)	58(69)		

Almost half of the participants (46.3%) did not know the peak season for CL transmission while 88(21.6%), 38(9.3%), 54(13.2%) indicated summer, autumn and spring respectively. Almost 19(4.7%) indicated that transmission was common all year round. More than half 237(58.1%) of the participants knew that sand flies transmit diseases compared to 189 (46.3%) who did not think that sand flies transmit any diseases. Among those who knew that sand flies transmit diseases, 153(37.5%) indicated that the fly can transmit CL. In addition, 24(5.9%) and 39(9.6%) believed CL was transmitted by mosquitoes and through direct contact with affected persons respectively. Almost 2.0% (1.96%) indicated bat’s urine or sputum were causes of CL. Other diseases thought to be transmitted by sand flies were diarrhoeal diseases 75(18.4%), other skin lesions 64(15.7%) and gastritis about 4(1.0%). With respect to clinical presentations, majority 295(72.3%) described CL based on the location and clinical appearance. About 192(47.1%) indicated disfiguring lesions as the main clinical presentation of CL, 137(33.6%) as painful lesions, 9(2.2%) as painless lesion. Another 55(13.5%) did not know of any symptoms of CL. Over half 225(55.1%) received information about CL from the community and 97(23.8%) did not have sources of information about the disease. Other sources were schools (health education) 61(14.9%) and community health education 6(1.5%).

### Attitude towards of CL

Over half (51.7%) of the participants with tertiary education had seen patients with CL followed by those with primary level education 106(29.6%). Among participants who had seen CL, 337 (82.6%) indicated that the disease was a serious problem in their area. Cosmetic consequences as reasons for seriousness of the disease was stated by 275 (67.4%), disability 190(46.6%) and 74(18.1%) indicated stigma. However, there was no statistically significant association (*p* > 0.025) between the level of education and attitude towards CL except for the knowledge on preventive measures which significantly associated (*p* = 0.014) with level of education (Table 3).

### Attitude towards treatment and treatment options for CL

Majority 294(82.1%) of rural residence had seen patients with CL compared to 64(17.9%) of the urban residents. However, no statistical difference (*P* = 0.269) was observed whether the participants had heard about sand fly. While majority of the participants were concerned that CL is a serious condition less than half 55(27.5%) of the urban residents believed that CL is treatable compared to 145(72.5%) among rural residents (*P* < 0.000). Over half (68.3%) of the participants indicated that most patients with CL received traditional medicines from traditional healers and traditional medicines as the best treatment option was indicated by 209(51.2%) who believed so because of easy accessibility compared to 114(27.9%) who indicated modern medicines as their best treatment option. The reasons given for the choice of modern treatment as the best treatment option included easy accessibility 22(5.4%), the only option 53(13.0%) and short duration of treatment 26(6.4%). Only 50(12.3%) knew names of modern medicines for treating CL among which 33(8.1%) indicated that medicine could be obtained from hospitals and the remaining 4.2% from health centres. There was no statistically significant association (*P* > 0.025) between the level of education and attitude towards treatment of CL (Table 3) but attitude towards treatment of CL significantly (*P* < 0.025) associated with location with rural residents showing more concerns (Table 4).

### Knowledge of preventive measures of CL

Among the total participants, 300(73.5%) selected one or more preventive measures while 108(26.5%) did not know of any preventive measures against the infecting agent. Majority of the participants 171(41.9%) and 129(31.6%) indicated hygiene and bed nets respectively. Health education was stated by 76(18.6%) as necessary in the prevention of CL. preventive measures. For prevention of sand fly bites, majority 149(36.5%) did not know of any prevention methods while only 53(12.9%) and 37(9.1%) considered hygiene and closing of windows and doors as prevention methods from sand fly bites (Table 5).

**Table 5:** Practice of respondents towards prevention of CL in northwest, Ethiopia

Practice description	Frequency	Percent (%)
What are the prevention measures for cutaneous leishmaniasis?		
Health education	76	18.6
Hygiene	171	41. 9
Bed net	129	31.6
Insecticide	58	14.2
I don’t know	108	26.5
Which sand fly prevention methods do you know?		
Ointment	5	1.2
Insecticides	53	12.9
Closing door and windows	37	9.1
Hygiene	122	29.9
Other	42	10.3
I don’t know	149	36.5

## Discussion

In 2014, the World Health Organisation stressed the serious and increasing threat of vector-borne diseases in the world including leishmaniasis with a slogan “*Small bite, big threat*” [18]. Hence, there is a need for intensified research to understand the knowledge, attitude and practices of communities living in the endemic areas about CL. We found most participants had seen CL and knew its clinical manifestations. Similar findings had earlier been reported in Ochello, southern Ethiopia [19]. Our findings on the community’s knowledge of CL are however better than reports from India where respondents recognised pictures of CL shown to them but did not have any lay perceptions about the disease [20, 21]. These differences could be due to differences in the approach to socio-cultural factors and prevention strategies between countries.

### Knowledge on CL and its transmission

Knowledge on CL and involvement of sand flies had a significant correlation with implementation of sand fly control measures in Guatemala [22] and Colombia [23]. Knowledge on the disease resulted in behaviour directed towards its prophylaxis and treatment [24]. We observed gaps in the knowledge on the transmission of the disease and still there are people in the community who believe that the parasite is transmitted via mosquitoes and through direct contact with infected persons. Though our findings are lower than 37.5 and 59.7% in a study in India where respondents claimed the role of mosquitoes in the transmission of CL and direct transmission from one person to another through direct contact respectively [20], such beliefs need to be addressed and correct information provided to communities in order to reduce potential for stigmatisation of affected individuals.

The transmission cycle of Leishmania exhibits characteristics that are particular to each endemic area therefore, does not allow extrapolation of data from one region to another [25]. In this study some significant proportions of the respondents did not have the correct knowledge on the peak season and time when the insects (sand fly) bite. These results are consistent with the report in Pakistan where 54.8% of the participants were unaware of the time when the insect bites and 24.8% believed that the peak season for sand fly bites was summer [14]. In most endemic areas, the transmission of the disease is almost throughout the year peaking after rainy season. Understanding the period of the year and the period of the day when transmission is highest is important for communities to prepare themselves by putting in place necessary preventive measures.

### Perception and attitude towards CL and its treatment

Studies have indicated a direct relationship between the knowledge of the population at risk of a disease and preventive measures [10]. In this study, majority of the participants believed that CL is a serious condition and were concerned about the cosmetic and disability consequences of the diseases. These findings are higher than those reported in Paraguay where only 10% of the participants perceived that CL was a problem [26]. Residence in rural areas significantly correlated with having seen a patient with CL, knowledge, attitude and practices about CL. Applying the Integrated Behaviour Model [27] which is grounded on the theory that an individual’s intention to engage in a behaviour is influenced by his/her attitude (*experiential*) towards the behaviour, our study indicates that high incidence, serious consequences of the disease and socioeconomic are key factors which have influenced concerns among rural residents about CL as presented with a significant association between rural residence and CL.

Usually CL heals spontaneously but in delayed treatment the disease can lead to serious tissue damage, secondary infection, disfiguring scar formation, impaired function and psychosocial consequences including depression. Delay in seeking treatment is associated with negative perceptions and attitude towards treatment options and lack of or inadequate information access about CL treatment have influence on the people’s treatment seeking behaviour [15, 19]. In many developing countries traditional healers play an important role in the delivery of health care and majority of the populations depend on them for most of their ailments [28]. We found that respondents believed CL is treatable and traditional medication was the best treatment option over modern treatment. Participants expressed positive experiential attitude towards traditional medicine because of its easy accessibility and expressed negative experiential attitude towards modern treatment because it is not easily accessible and the places where modern medicines can be accessed are far from the communities. A report on the review on various countries have suggested that traditional healers if properly trained can contribute positively to the primary health care teams and recommendations were made to take advantage of traditional healers as valuable resources to provide relevant and appropriate information and timely treatment in order to reduce consequences of delayed treatment of CL [29]. The government and other stakeholders involved in the provision of health services in Ethiopia should therefore bring the services closer to the affected populations by introducing mobile clinics, constructing health facilities in the endemic areas, making medications available at no or reduced cost and raising awareness of the community of the advantages of using modern treatment. Similarly, providers of health care in the affected areas should engage with traditional healers and determine effective ways of integrating traditional healers in the provision of care.

### Perceptions on CL prevention and control

Most participants believed that CL is preventable and the most preferred preventive approach was personal hygiene. Education on CL and its consequences has been reported to be cost-effective and improves uptake of preventive measures [30]. In that regard, primary prevention like health education should be given priority through identification of population groups at risk including those involved in activities without using protection either by insecticides or clothing [31, 32]. On the use of bed nets, communities need to be educated that since sand flies are much smaller than mosquitoes impregnated bed nets with much smaller maze should be used. In addition, communities should be informed on how to reduce the natural reservoirs of Leishmania*.*

The epidemiology of leishmaniasis is influenced by several factors including suitable vectors, environmental conditions, socio-economic status, demographic and human behaviours [33, 34]. Poor housing, migration in search for employment, deforestation, immunosuppressive conditions like HIV and AIDS and malnutrition are some of the risk factors implicated on the prevalence of leishmaniasis [35, 36]. Like in many developing countries, these factors are also prevalent in Ethiopia. The country therefore, should put in place coordinated prevention, control and eradication programmes that would reduce public health and socio-economic impact of the disease to communities in endemic areas. The media including television, radio stations, printed media and political platforms should intensify raising community awareness on CL and because of the strong beliefs communities have on traditional medicine, the government should attempt to authenticate traditional healers and research should be carried out to determine the efficacy and safety of traditional medicines used to treat CL.

## Limitations

Limitations of this study include limited number of open ended question which would have helped the respondents to add more information to the provided questions, and the findings may not necessarily be generalizable to the whole country because of differences in the population dynamics like environment, economy, and educational status. In addition, immunosuppression and co-morbidity could influence the manifestation and severity of the diseases.

## Conclusion

This study has shown the knowledge gaps about CL which have contributed to negative experiential attitude and perceived control towards modern medicines. Inaccessibility and long distances from where modern treatment for CL is provided have reinforced positive experiential attitude towards traditional medicine. This information is valuable and should be used as indicators for awareness campaigns, health education, health promotion, future research on the disease and for designing appropriate policies to guide the government and community efforts against CL in endemic communities. School should feature prominently in the campaign by developing training curricula that provide children and communities with correct information on CL.

## Data Availability

The datasets used and/or analysed during this study are available from the Corresponding Author on reasonable request.
